# Advancing clinical integration of circulating tumor cells: Outcomes of an international expert consensus

**DOI:** 10.1016/j.jlb.2025.100452

**Published:** 2025-12-27

**Authors:** Massimo Cristofanilli, M. Jose Serrano

**Affiliations:** a Department of Medicine, Division of Hematology-Oncology, Weill Cornell Medicine, New York, NY, USA; b Department of Pathology, Virgen de las Nieves University Hospital, Granada, Spain; c Institute for Biomedical Research (ibs. GRANADA), Granada, Spain

Circulating tumor cells (CTCs) was the first liquid biopsy tests in solid tumors. Several prospective clinical studies conducted for over two decades demonstrated their prognostic value and the ability to measure tumor dynamics in real time [1], [2]. Their value as biomarkers in metastatic disease is well documented, and platforms such as CellSearch® obtained regulatory clearance in breast, prostate, and colorectal cancer [1], [3].

In spite of such results, their use in routine clinical practice remains limited. There are several reasons to justify such limitation: evidence varies across tumor types, workflows are not standardized, and in many settings, clinical utility has not yet been demonstrated in a way that directs treatment decisions.

To address these issues, the International Society of Liquid Biopsy (ISLB) organized an expert workshop in Granada, Spain, on May 24–25, 2024. The purpose of this meeting was straightforward: to review the evidence accumulated to date, identify areas where CTCs are ready for clinical use, and define the steps needed to expand their role as predictive biomarker in solid tumors. The discussions in Granada guided the structure of the subsequent Delphi-based consensus process and directly informed the final recommendations [4].

The consensus confirms that CTCs have a defined clinical role in metastatic breast cancer and metastatic prostate cancer [4]. In these settings, CTC enumeration is a validated prognostic marker, and changes in CTC levels during treatment can help evaluate therapeutic benefit. In metastatic castration-resistant prostate cancer, detection of AR-V7 in CTCs may assist in selecting between androgen receptor–targeted therapies and taxanes [5].

Outside these contexts, CTC enumeration remains mainly a prognostic tool [4]. In colorectal and lung cancer, for example, CTC counts correlate with outcomes, but current data do not yet support treatment decisions based on CTC analysis. Prospective trials focused on clinical utility will be required to move the field forward immunotherapies, where actionable biomarkers are essential for patient selection.

A central point emerging from the consensus is the shift from counting CTCs to molecular characterization. Phenotypic and molecular profiling offers the possibility to identify therapeutic targets, monitor resistance, and understand clonal evolution during treatment. This direction aligns naturally with the increasing use of targeted therapies and antibody–drug conjugates, where *real-time* biomarker information is needed.

The panel also highlights the complementary roles of CTCs and circulating tumor DNA (ctDNA). While ctDNA reflects the genetic landscape of the tumor, CTCs allow for multi-level characterization at the single-cell level, including protein expression and functional properties [4]. Integrating both analytes may be particularly valuable in minimal residual disease monitoring and early detection of treatment resistance.

To support broader clinical adoption, the following priorities are emphasized:

• Standardize sample collection and analysis procedures.
• Conduct prospective multicentre clinical trials designed to demonstrate impact on patient management.
• Support development of more sensitive and flexible CTC technologies.
• Provide education and training to ensure appropriate interpretation.

The ISLB is well positioned to coordinate these efforts, given its focus on liquid biopsy technologies and its international membership across laboratory, clinical, and translational research settings.

This consensus is intended not as a conclusion, but as a direction of travel. The evidence supporting CTCs is substantial in some diseases and promising in others. The next step is to demonstrate, through well-designed clinical studies, how CTC analysis can guide therapy and improve outcomes. As liquid biopsy continues to expand, CTCs are likely to become an integral component of precision oncology, complementing ctDNA and tissue-based diagnostics.

## Ethical approval/patient consent

This article is an editorial and does not involve human participants, clinical data, or identifiable patient information; therefore, ethical approval and patient consent are not required.

## Funding

The authors declared no specific grant for this editorial from any funding agency in the public, commercial, or not-for-profit sectors.

## Declaration of competing interest

The authors declare that they have no known competing financial interests or personal relationships that could have appeared to influence the work reported in this paper.

## References

[1] Cristofanilli M., Budd G.T., Ellis M.J. (2004). Circulating tumor cells, disease progression, and survival in metastatic breast cancer. N Engl J Med.

[2] Gaforio J.J., Serrano M.J., Sanchez-Rovira P., Sirvent A., Delgado-Rodriguez M., Campos M., de la Torre N., Algarra I., Dueñas R., Lozano A. (2003). Detection of breast cancer cells in the peripheral blood is positively correlated with estrogen-receptor status and predicts for poor prognosis. Int J Cancer.

[3] de Bono J.S., Scher H.I., Montgomery R.B. (2008). Circulating tumor cells predict survival benefit in metastatic castration-resistant prostate cancer. Clin Cancer Res.

[4] Nicolò E., Reduzzi C., Pierga J.Y. (2025). International expert consensus on the clinical integration of circulating tumor cells in solid tumors. Eur J Cancer.

[5] Antonarakis E.S., Lu C., Wang H., Luber B., Nakazawa M., Roeser J.C., Chen Y., Mohammad T.A., Chen Y., Fedor H.L., Lotan T.L., Zheng Q., De Marzo A.M., Isaacs J.T., Isaacs W.B., Nadal R., Paller C.J., Denmeade S.R., Carducci M.A., Eisenberger M.A., Luo J. (2014). AR-V7 and resistance to enzalutamide and abiraterone in prostate cancer. N Engl J Med.

